# Proactive psychological first aid in mass-casualty recovery: a theory-driven case analysis of the SIX Cs model

**DOI:** 10.3389/fpsyg.2026.1875015

**Published:** 2026-06-11

**Authors:** Einav Levy, Moshe Farchi

**Affiliations:** 1 Department of Social Work, The Research Center for Innovation in Social Work, Tel Hai Qiryat Shmona University of the Galilee, Israel; 2 The Israeli School of Humanitarian Action, Tel Aviv, Israel

**Keywords:** HAMAS – Israel war, mass-casualty body recovery, proactive cognitive structuring, psychological first aid (PFA), SIX Cs model

## Abstract

This article presents a theory-driven case analysis of a proactive application of the SIX Cs Model of a proactive application of the SIX Cs Model of Psychological First Aid (PFA) during a mass-casualty body recovery mission conducted on October 8, 2023, in Kibbutz Be'eri, Israel, following the Hamas attack of October 7, 2023. Unlike conventional reactive PFA, which is delivered in response to acute distress, the present case illustrates a prospective implementation in which cognitive structuring, task rehearsal, role assignment, and sequenced closure were employed before, during, and after direct exposure to mass fatality. The intervention was led by a trained military team commander (the first author of this manuscript) who systematically operationalized the SIX Cs components (i.e. *Cognitive*-internal mental structuring; *Communication*- explicit, task-oriented information delivery; *Challenge, Control, Commitment*, and *Continuity*) across three phases: pre-mission briefing, on-site execution, and post-mission After-Action Review (AAR). The case demonstrates how structured pre-exposure cognitive organization may help counteract anticipatory helplessness, reduce perceptual narrowing, and sustain *cognitive* engagement throughout sustained traumatic exposure. Possible neurological and psychological mechanisms underlying each component are discussed in relation to observable behavioral outcomes. The case offers a theoretical and conceptual basis for exploring the applicability of the SIX Cs Model in varied operational contexts, and suggests that proactive Psychological First Aid may constitute a distinct intervention modality warranting further systematic investigation.

## 1 Introduction

Mass-casualty events impose extreme psychological demands on responders, whose exposure to human remains, odor, and the aftermath of violence creates conditions of acute and sustained traumatic stress. While Psychological First Aid (PFA) has been increasingly recognized as an evidence-informed approach for supporting individuals during and after crisis events, most existing frameworks are designed for reactive application, delivered after distress has emerged rather than before distress arises. The SIX Cs Model (Farchi et al., 2018) represents a structured cognitive framework that targets core features of the Acute Stress Response (ASR), including helplessness, perceptual narrowing, cognitive disorganization, and impaired executive functioning. The SIX Cs Model's components—*Cognitive* (internal mental activation and structuring of thought under stress), *Communication* (explicit, task-oriented information delivery that renders the situation comprehensible and actionable), *Challenge, Control, Commitment*, and *Continuity*, provide a systematic protocol for restoring prefrontal engagement and adaptive behavior during extreme conditions.

The Hamas attack of October 7, 2023, in Israel resulted in the deaths of over 1,200 civilians and created an unprecedented mass-casualty event requiring immediate deployment of recovery teams. On October 8, 2023, the author deployed with a structured military team to Kibbutz Be'eri, one of the most severely affected communities, to conduct body identification, containment, and evacuation operations in collaboration with ZAKA (Zihuy Korbanot Ason—Hebrew for Disaster Victim Identification), an Israeli volunteer-based emergency response organization specializing in the dignified recovery and handling of human remains. Rather than awaiting on-site instructions from coordinating organizations, the conventional operational approach, the team commander implemented a pre-exposure cognitive structuring protocol grounded in the SIX Cs Model. This proactive application of PFA principles constitutes the subject of the present theory-driven case analysis.

The present article describes the implementation of the SIX Cs components across three mission phases, explores possible connections between observed behavioral and functional outcomes and their hypothesized neurological and psychological mechanisms, and discusses the conceptual implications for first-responder Psychological First Aid training and deployment protocols.

### 1.1 The SIX Cs model and the ASR

The SIX Cs Model was developed as a structured cognitive framework for Psychological First Aid, targeting core features of the ASR such as cognitive disorganization, perceptual narrowing, helplessness, and impaired executive functioning (Farchi et al., 2018, 2024). *Cognitive* activation re-engages internal executive processes, including attention, planning, and mental organization, that are disrupted by acute stress. Communication, which is both distinct from and operationally coupled with *Cognitive* activation, refers to the explicit, task-oriented verbal delivery of structured information that renders the situation comprehensible and actionable for the individual. Together, *Cognitive* activation provides the internal mental scaffolding, while *Communication* provides the interpersonal vehicle through which that scaffolding is constructed and maintained. *Challenge* introduces brief, achievable tasks that disrupt freezing, promote goal-directed action, and create immediate experiences of competence. *Control* restores agency through simple, meaningful choices, enhancing functional capacity and self-efficacy while reducing helplessness; because choosing between options is a logical process, the *Control* component may also support cognitive dominance over automatic stress responses during acute stress. *Commitment* provides continuous, reliable presence that reduces loneliness and sustains the individual's engagement and cooperation with the helper. Continuity structures the sequence of events, reducing fragmentation and preventing maladaptive memory encoding.

Together, these components support autonomic regulation and functional restoration during acute stress, with accumulating evidence of reductions in anxiety and improved indices of physiological regulation and resilience following SIX Cs-based interventions (Farchi et al., 2018; Farchi and Shlezinger, 2026; Thayer et al., 2012).

### 1.2 Proactive application of the SIX Cs: a theoretical extension

The original formulation of the SIX Cs Model was developed primarily for reactive implementation, that is, for application to individuals already experiencing acute distress (Farchi et al., 2018). However, the cognitive and psychological logic underlying each component suggests that identical mechanisms may operate prospectively, before traumatic exposure, to reduce vulnerability to ASR. Anticipatory helplessness, the subjective sense that one will be unable to manage an impending stressor, may share features with experienced helplessness, including elevated stress reactivity, reduced executive engagement, and heightened emotional reactivity (Maier and Seligman, 2016). Proactive cognitive structuring that introduces task clarity, rehearsal, and role definition prior to exposure may therefore modulate these same pathways, functioning as inoculation against the functional collapse that characterizes ASR.

This conceptualization shares certain features with stress inoculation training, particularly the preventive, pre-exposure orientation and the use of structured preparation to reduce uncertainty and build a sense of readiness. However, the present protocol differs from stress inoculation training in important respects: whereas stress inoculation training is designed to build resilience through psychoeducation about stress reactions, skills acquisition, and graduated exposure to stressors, the proactive SIX Cs application described here focuses on cognitive briefing, procedural rehearsal, task-oriented scaffolding, and structured closure, mechanisms oriented toward reducing anticipatory helplessness and sustaining executive functioning during a specific, time-bounded operational mission. The conceptual overlap between these approaches, particularly their shared preventive logic, is noted, while their distinct mechanisms and goals are acknowledged (Meichenbaum, 2019; Driskell et al., 2018). The present work is a theory-driven case analysis grounded in a real-time mass-casualty response operation. The case is examined through the lens of the SIX Cs framework, with operational decisions and observed behavioral outcomes mapped onto model components and their hypothesized neurological and psychological mechanisms.

The application of the SIX Cs in this case was further distinguished by its extension into the post-mission phase. The evening After-Action Review (AAR) conducted following the body recovery operation operationalized *Continuity* and *Commitment* in a reflective mode, supporting coherent memory encoding, reducing emotional isolation, and constructing a shared narrative of mission success. This three-phase model (proactive preparation, structured execution, and cognitive closure) represents a novel elaboration of the SIX Cs framework as a comprehensive responder support protocol.

It is worth noting explicitly how the proactive SIX Cs framework described here differs from two superficially similar approaches: stress inoculation training and standard mission rehearsal. Stress inoculation training builds resilience through psychoeducation about stress reactions, graduated exposure to stressors, and the acquisition of generalized coping skill, a process typically requiring sustained training across multiple sessions. The proactive SIX Cs protocol, by contrast, operates within the immediate pre-mission window, targeting specific psychological mechanisms of ASR vulnerability, anticipatory helplessness, cognitive disorganization, perceptual narrowing, behavioral freezing, relational isolation, and memory fragmentation, through structured briefing, role assignment, procedural rehearsal, and explicit sequencing, without requiring prior generalized stress exposure. Standard mission rehearsal, for its part, focuses primarily on task competence and procedural familiarity, without a theoretically specified account of the psychological mechanisms being addressed or a structured post-exposure phase oriented toward adaptive memory encoding. The SIX Cs protocol is distinguished by its component-level psychological rationale, its explicit three-phase structure extending from pre-exposure preparation through post-mission cognitive closure, and its grounding in a coherent theory of ASR and its remediation. These distinctions position the proactive SIX Cs framework as a theoretically specified psychological intervention rather than an elaborated operational briefin, while acknowledging, as noted elsewhere in this manuscript, that empirical confirmation of these distinctions remains an important direction for future research.

### 1.3 Case description and analytic approach

The present work is a single, theory-driven case report based on a real-time mass-casualty response operation. The case is reported by the team commander who designed and implemented the intervention protocol described herein. The analysis links operational decisions and behavioral outcomes to SIX Cs components and possible neurological and psychological mechanisms.

#### 1.3.1 Context and setting

The Hamas attack of October 7, 2023, in Israel resulted in the deaths of over 1,200 civilians and created an unprecedented mass-casualty event requiring immediate deployment of recovery teams. Kibbutz Be'eri was among the most severely affected communities: the attack involved the systematic killing of civilians in their homes, including acts of extreme violence against men, women, children, and the elderly, resulting in scenes of mass fatality that were exceptional in their scale, visibility, and psychological intensity even relative to other mass-casualty environments. The conditions encountered by recovery teams included direct, prolonged exposure to large numbers of human remains in states of significant traumatic injury, in domestic and communal settings, compounding the psychological demands beyond those typically associated with conventional first-responder or mortuary affairs operations. On October 8, 2023, the author deployed with a structured military team to Kibbutz Be'eri, one of the most severely affected communities, to conduct body identification, containment, and evacuation operations in collaboration with ZAKA.

The team comprised a combat reserve unit that had been mobilized in response to the October 7 attack as part of the broader emergency military call-up. The unit was an organic, long-standing team with years of shared operational experience in combat and defensive missions; however, the recovery, handling, and containment of human remains fell entirely outside the team's designated role and prior training. The members had not previously performed, nor been trained for, body recovery operations of any kind. This context is clinically significant: the team was not a specialized forensic or mortuary affairs unit, but a cohesive combat team suddenly confronted with a qualitatively unfamiliar and psychologically extreme task.

Upon arrival at a staging area, the team encountered ZAKA representatives who indicated that procedural guidance would be provided on-site, at the location of each body. Rather than proceeding according to this conventional approach, the team commander introduced a pre-exposure structured briefing protocol grounded in the SIX Cs Model.

#### 1.3.2 Pre-mission structured briefing

Before approaching the body recovery sites, the team assembled for a structured cognitive briefing. The following seven operational principles were established and rehearsed:

1. Organized assignment of pairs and triads before arrival at body locations.
2. Thorough rehearsal of body-bagging procedures by all pairs, including all possible operational variants.
3. Body handling technique: grip, transfer, and bagging procedures.
4. Explicit sequencing of actions to be performed upon arrival at each body location.
5. Defined site management protocol including spatial organization and team movement.
6. Communication structure with the ZAKA site manager.
7. Odor management strategies.

#### 1.3.3 On-site execution and administrative closure

Teams were deployed in accordance with the briefed structure. The team commander maintained continuous presence on-site, monitoring team functioning and providing ongoing structured Communication in the form of precise, task-oriented guidance, to sustain *Cognitive* activation as required (See Table 1). Upon completion of the body recovery operation, a structured administrative closure was conducted before departure from the site. This closure was explicitly cognitive and procedural in character, providing a defined endpoint to the operational phase of the event.

**Table 1:** Integration of the SIX Cs components in the proactive body recovery protocol.

SIX Cs component	Role in Pre-mission structuring	Possible neurological/psychological mechanism	Functional outcome
*Cognitive*	Pre-mission briefing imposed a clear mental structure on the task: roles were assigned, action sequences were defined, and all procedural variants were rehearsed mentally before exposure. This organized the team's internal cognitive map of the operation.	May reduce cognitive disorganization and perceptual narrowing; may support working memory under stress. Structured mental preparation reduces anticipatory helplessness and builds cognitive scaffolding for organized behavior Arnsten (2015).	Enabled each team member to hold a clear internal representation of the mission; reduced confusion and decision fatigue during exposure.
*Communication*	The briefing was delivered using precise, unambiguous language; information was structured sequentially; roles and responsibilities were named explicitly. The commander continued to deliver structured verbal communication on-site throughout the operation. Note: Communication is the interpersonal vehicle through which Cognitive activation is constructed and sustained.	Reduces ambiguity and supports orientation and predictability; reinforces perception that the situation is comprehensible and manageable (Antonovsky, 1987). Communication externalizes cognitive structure, making it jointly accessible to all team members.	Aimed to restore shared orientation before exposure; may have enabled the team to execute tasks more coherently despite high emotional arousal; appeared to maintain alignment throughout the operation.
*Challenge*	Rehearsal of body-bagging procedures and simulation of all possible scenarios before arrival at the site.	May reduce sympathetic arousal and improve motor planning under anticipated stress. Structured rehearsal builds perceived self-efficacy Bandura (1997) and transforms abstract threat into concrete, manageable steps.	May have counteracted freezing potential; appeared to promote goal-directed action; aimed to build a sense of competence prior to exposure.
*Control*	Each team member was assigned specific, bounded responsibilities. Choices within tasks were explicitly defined.	May enhance volitional capacity and reduce helplessness. Role assignment restores personal agency, shifting orientation from passive victim to purposeful actor within a structured framework Langer (1983); Seligman (1975).	May have reinstated a sense of agency; aimed to reduce anticipated helplessness; appeared to support decision-making capacity during the mission.
*Commitment*	Team leader maintained continuous presence; explicit norms of mutual support were established before departure.	May reduce stress reactivity via co-regulation. Explicit mutual support norms and leader presence counter isolation under trauma exposure; social support is among the strongest protective factors against acute stress responses (Ozer et al., 2003).	Appeared to sustain team cohesion and cooperation; aimed to maintain motivation and responsiveness throughout the mission.
Continuity	Sequence of actions was explicitly structured: pre-mission briefing → rehearsal → on-site protocol → administrative closure → team AAR.	May reduce memory fragmentation and lower risk of intrusive recollections. Explicit temporal structuring provides a coherent narrative frame supporting meaning-making and reducing chaos under traumatic exposure (Park, 2010).	May have enabled more coherent processing of the event; aimed to reduce confusion and support adaptive memory encoding and post-event closure.

The following table presents the systematic mapping of SIX Cs components onto the pre-mission structuring protocol, including possible neurological and psychological mechanisms and functional outcomes.

#### 1.3.4 Evening after-action review (AAR)

On the evening following the mission, the full team gathered for a structured group AAR. This session comprised: (1) a sequential review of the day's events; (2) structured and cognitively framed sharing of individual experiences and emotional responses; (3) explicit construction of a continuous, coherent memory narrative of the mission; and (4) affirmation of the team's successful execution of the mission despite its psychological complexity (see Table 2). The AAR was designed to support adaptive memory encoding, reduce emotional isolation, and facilitate cognitive closure.

**Table 2:** Mission protocol phases, actions, and Six Cs mapping.

Phase	Action/communication	SIX Cs component	Functional rationale
Pre-mission (off-site)	Team leader assembled pairs and triads; assigned roles; reviewed full task scope.	*Cognitive*; *communication*; *control*	Aimed to establish a clear cognitive map; may have reduced anticipatory confusion; intended to promote agency.
Pre-mission (off-site)	Full rehearsal of body-bagging procedure including all variant scenarios (partial remains, odor, positioning).	*Challenge*; *cognitive*	Aimed to build procedural competence; may have reduced freezing risk.
Pre-mission (off-site)	Explicit protocol walkthrough: sequence of actions upon arrival at each body location.	Continuity; *control*	Aimed to establish temporal and procedural coherence; may have reduced decision fatigue.
Pre-mission (off-site)	Defined site management procedures: coordination with ZAKA, movement on site, communication norms.	*Cognitive*; *communication*; *commitment*	Aimed to clarify roles; may have reduced ambiguity; intended to reinforce relational structure.
Pre-mission (off-site)	Briefing on body handling techniques (grip, transfer) and odor management strategies.	*Challenge*; *communication*; *cognitive*	Aimed to translate abstract threat into manageable technical skill.
On-site arrival	Structured gathering before approaching body locations; task reassignment confirmed.	*Communication*; *cognitive*; *continuity*	Aimed to re-anchor the cognitive frame; may have maintained procedural continuity.
On-site	Teams executed body retrieval according to rehearsed protocol; team leader-maintained presence.	All SIX Cs	Aimed to preserve executive functioning; appeared to sustain task completion.
On-site closure	Administrative closure conducted coherently and cognitively before departure.	*Continuity*; *cognitive*; *communication*	Provided a structured endpoint; aimed to reduce the risk of fragmented memory encoding.
Evening AAR	Full team AAR: event review, structured emotional sharing, continuous memory construction, mission success affirmation.	*Commitment*; *continuity*; *cognitive*; *communication*	Aimed to integrate the experience cognitively; may have reduced isolation; sought to construct a shared narrative and facilitate adaptive processing.

The following table maps each phase of the mission to the corresponding operational actions, SIX Cs components, and functional rationale.

## 2 Post-event observations: narrative integration and long-term team functioning

Informal observations gathered through non-structured conversations with team members in the days and weeks following the mission offer a preliminary, unsystematic, and exploratory account of experiences that may be conceptually consistent with the hypothesized aims of the proactive SIX Cs protocol. These observations are not presented as evidence of intervention efficacy but as narrative illustrations of how team members appeared to relate to the structured preparation they received. Team members spontaneously described the mission as a psychologically demanding experience, yet one that they perceived as having been managed effectively. Specifically, individuals referenced the pre-exposure structuring, the sense of clarity and procedural certainty provided before contact with human remains, the administrative and cognitive closure of the event, and the cohesion of the team's coordinated functioning as factors that contributed to a sense of agency and competence in the face of an extreme stressor. Rather than describing the event primarily in terms of distress or disruption, participants framed it as a meaningful reference point within their personal and collective narratives, one that confirmed their capacity to function effectively under conditions of extreme psychological demand. This pattern may reflect the consolidation of a coherent, efficacy-affirming event narrative, consistent with theoretical accounts of post-traumatic growth and meaning-making in responder populations (Tedeschi and Calhoun, 2004; Park, 2010).

Observations gathered approximately 2 years after the event suggest a degree of longitudinal stability in this pattern. In informal conversations conducted within the context of the ongoing reserve service that followed the October 7 attack, team members continued to reference the Be'eri mission as a foundational element of their individual and collective identity as a unit. The event was described not as a source of impairment or avoidance, but as a formative experience that reinforced a shared sense of professional competence, mutual trust, and mission coherence. Notably, despite the sustained operational demands and documented attrition pressures associated with prolonged reserve duty during wartime, the team has remained intact and operationally functional as a unit. While causal attribution is not possible within the constraints of a single case report, this observation is consistent with the proposition that structured proactive PFA may contribute not only to acute stress regulation but also to longer-term cohesion and resilience at the team level. These post-event observations, though informal, unsystematic, and subject to significant retrospective bias, are conceptually consistent with the proposition that the proactive SIX Cs structure may have contributed to a shared efficacy narrative, a collective sense that the team acted with competence and purpose under extreme conditions. This interpretation remains speculative and is offered as a conceptual frame for future inquiry rather than as a demonstrated outcome of the intervention.

## 3 Discussion

The present case provides an illustration of the SIX Cs Model applied proactively, before acute exposure, in a mass-casualty first-responder context. The pre-mission briefing systematically targeted the principal mechanisms of ASR vulnerability: anticipatory helplessness was addressed through role assignment and *Control*; cognitive disorganization was pre-empted through structured *Cognitive* activation, the internal mental scaffolding, and through precise Communication that rendered the structure externally accessible to the team; freezing risk was reduced through behavioral rehearsal (*Challenge*); relational isolation was countered through Commitment norms; and memory fragmentation was mitigated through the establishment of *Continuity* across mission phases. It is important to note that while *Cognitive* and *Communication* are analytically distinct components, the former operating at the level of internal mental organization, the latter at the level of interpersonal information delivery, they are operationally coupled: structured Communication is the primary mechanism through which *Cognitive* activation is induced and sustained in a team setting.

From a cognitive and psychological perspective, the structured pre-exposure protocol is consistent with a form of proactive executive priming (Arnsten, 2015). By engaging planning, sequencing, role definition, and rehearsal before the encounter with traumatic stimuli, the intervention may have elevated the threshold for stress-driven disruption of organized behavior. Evidence from stress inoculation research supports the principle that controlled anticipatory preparation can be interpreted as attenuating physiological stress responses and sympathetic nervous system activation during subsequent exposure (Driskell et al., 2018; Meichenbaum, 2019). The structured nature of the briefing, grounded in the SIX Cs framework, is consistent with this principle and offers a degree of precision and reproducibility that may support future replication.

The post-mission AAR represents an equally significant application of SIX Cs principles. Continuity was extended beyond the operational phase into the reflective phase, providing a structured narrative container for the team's experience. *Commitment* was maintained through the shared group format, reducing the risk of individual isolation that is associated with secondary traumatic stress in responders (Greinacher et al., 2019). The cognitive framing of emotional sharing, explicitly structured rather than unguided, is consistent with evidence that deliberate, organized processing of traumatic material supports adaptive memory consolidation and reduces intrusive symptomatology (Brewin, 2018; Haubrich and Nader, 2016).

A central implication of this case is that the SIX Cs Model is not limited to reactive application with primary victims of acute threat but is adaptable as a proactive protocol for responders facing anticipated traumatic exposure. The three-phase structure documented here- pre-mission cognitive preparation, structured on-site execution, and post-mission cognitive closure, constitutes a replicable model for responder PFA that extends the original SIX Cs framework. This extension is particularly relevant in mass-casualty and prolonged-operations contexts, where repeated exposure accumulates over time and where the prevention of acute and chronic stress responses in responders is of operational and ethical importance.

Several features of this case warrant particular attention. First, the proactive protocol was implemented against the conventional approach offered by the coordinating organization (ZAKA), which relied on on-site, reactive instruction. The team commander's decision to implement pre-exposure structuring represents an active clinical judgment grounded in PFA theory. Second, the protocol was implemented successfully despite the extreme and unprecedented nature of the operational context, the largest mass-casualty event in Israel's history, suggesting that the SIX Cs framework is robust to conditions of maximum environmental stress. Third, the full three-phase protocol was implemented without professional mental health personnel present during the mission itself, indicating its potential for peer-leader implementation in resource-limited field settings.

A first key implication concerns the **timing of intervention**. The present case suggests that delivering structured cognitive preparation *before* exposure, rather than only during or after, may meaningfully alter the team's functional starting point. Rather than intervening once distress has already emerged, proactive structuring may shift the baseline from which responders engage with traumatic material, potentially raising the threshold at which stress responses interfere with effective functioning. This reframing of when PFA should be applied carries significant practical implications for responder preparation protocols and training curricula.

A second implication concerns the identity of the intervening agent. In the present case, the structured PFA protocol was designed and implemented not by a mental health professional, but by the **team commander**. This represents a potentially meaningful innovation warranting further investigation. Existing PFA frameworks are predominantly designed for delivery by trained clinicians or paraprofessionals operating in a support capacity. The present case demonstrates that a SIX Cs-grounded protocol can be operationalized by an operational leader with appropriate training, integrated naturally into mission preparation rather than delivered as a separate clinical intervention. This finding has direct implications for field settings where mental health professionals are unavailable, and supports the development of commander-led psychological resilience protocols as a distinct and viable modality within the broader PFA literature.

A third implication concerns the **goal of intervention**. Conventional PFA is often framed primarily in terms of reducing ASR once they have occurred. The proactive orientation of the present protocol suggests a fundamentally different aim: not merely the attenuation of distress, but the *prevention of helplessness from forming in the first place*. By establishing cognitive structure, role clarity, and procedural competence before exposure, the intervention may preempt the conditions under which learned helplessness and functional collapse emerge. This distinction, between reducing a response and preventing its formation, reframes the logic of responder PFA around anticipatory resilience rather than reactive recovery, and underscores the centrality of proactive application as a distinct and theoretically coherent intervention mode.

### 3.1 The SIX Cs protocol and the question of novelty: distinguishing proactive pfa from generic mission rehearsal

A reasonable question concerns the extent to which the present protocol represents a conceptually distinct contribution, rather than a theoretically reframed application of well-established principles of mission rehearsal. Mission rehearsal and operational briefing are standard features of military and emergency response preparation across many operational contexts, and their general utility in building task competence and reducing uncertainty is well documented (Driskell et al., 2018). It is therefore necessary to articulate explicitly what the proactive application of the SIX Cs Model adds beyond what generic mission preparation already provides.

The distinction rests on three interrelated dimensions. First, the SIX Cs protocol is not a generic preparation procedure but a theoretically specified psychological intervention: each component targets a discrete mechanism of ASR vulnerability, anticipatory helplessness, cognitive disorganization, perceptual narrowing, behavioral freezing, relational isolation, and memory fragmentation, grounded in empirically supported models of stress neuroscience and psychological regulation (Arnsten, 2015; Maier and Seligman, 2016). Standard mission rehearsal addresses task performance; the SIX Cs protocol explicitly addresses the psychological infrastructure that enables performance under conditions of maximal cognitive and emotional disruption. The goal is not merely procedural competence but the active priming of prefrontal regulatory capacity before traumatic stimuli are encountered. Second, the three-phase structure described in this case, proactive cognitive preparation, structured on-site execution, and post-mission cognitive closure, is not a feature of conventional mission preparation frameworks. The integration of a theoretically grounded After-Action Review oriented toward adaptive memory encoding, rather than purely operational debriefing, extends the intervention explicitly into the post-exposure phase in ways that generic mission rehearsal does not. Third, the model provides a component-level account of why each element of preparation functions as it does, making the intervention both replicable and modifiable across contexts. This specification is precisely what permits clinicians and commanders to adapt the protocol to novel settings without losing fidelity to its psychological rationale.

At the same time, this distinction must be stated with appropriate candor. The present case cannot demonstrate, through the evidence it provides, that the SIX Cs protocol produced outcomes superior to those that might have been achieved by a well-conducted conventional mission briefing. The structural overlap between the two approaches is genuine, and the theoretical arguments for SIX Cs specificity, though logically coherent, await empirical confirmation. Future comparative research, examining SIX Cs-grounded preparation against matched briefing controls across diverse operational contexts, is required to establish whether the theoretical specificity of the model confers measurable advantages over general preparatory practice.

### 3.2 Psychological risks, challenges, and cautions

A balanced evaluation of the present protocol requires explicit consideration of the psychological risks and challenges that may accompany its application, both at the level of the model and at the level of the specific implementation described in this case. These concerns do not invalidate the approach, but they are essential to name with precision in order to support responsible clinical translation and to avoid uncritical replication in contexts where conditions may differ substantially.

#### 3.2.1 Risks inherent to the model

Intensive cognitive structuring may function, in certain individuals, as intellectualization or emotional suppression rather than preparatory regulation, delaying rather than preventing distress (Gross, 1998). For individuals with elevated trait anxiety or prior trauma histories, explicit pre-exposure rehearsal may heighten anticipatory dread through sensitization rather than reduce it (Rachman, 1980). The model does not currently incorporate a mechanism for identifying such individual variation.

The team-based structure may generate implicit conformity pressure, inhibiting distress disclosure to preserve group cohesion—particularly salient in military contexts where emotional expression carries professional cost (Hoge et al., 2004). Role assignment may also create an illusory sense of preparedness: if actual conditions deviate substantially from those rehearsed, the collapse of this expectation may be more destabilizing than had no control expectation been established (Thompson et al., 1988).

#### 3.2.2 Risks specific to the present implementation

Peritraumatic dissociation—the partial disconnection from emotional or cognitive experience during exposure, is a well-documented acute response to mass-casualty environments (Marmar et al., 1994; Bernat et al., 1998). The structured protocol may have facilitated behavioral competence partly through dissociative rather than genuine regulatory processes, whose resolution could manifest as delayed distress or intrusive symptomatology. Without systematic measurement, this possibility cannot be excluded.

The commander simultaneously occupied two distinct roles- operational leader and psychological support figure generating inherent tensions. Team members may be reluctant to disclose distress to someone holding evaluative authority (Castro and Adler, 2011). Without a mental health professional present, there was no independent clinical capacity to identify acute psychiatric presentations, panic, dissociative episodes, or severe grief, that fall outside the scope of peer-leader PFA.

Post-event observations are subject to retrospective bias: positive narrative reconstruction of trauma may obscure subclinical distress not disclosed in informal conversations (Tedeschi and Calhoun, 2004). Long-term team cohesion may equally reflect the containing function of a close-knit military unit, suppressing individual distress through collective resilience norms (Shay, 1994). These considerations underscore the necessity of systematic, longitudinal outcome assessment in future research.

## 4 Limitations

Several limitations should be acknowledged. This is a single theory-driven case analysis authored by the team commander, who designed and implemented the intervention, and as such it is subject to observer bias, role-based interpretation, and the well-documented tendency toward positive retrospective reconstruction of consequential events. The authors are aware that this dual role, as both intervening agent and primary analyst, constitutes a significant methodological limitation that independent replication would be required to address.

Critically, no systematic quantitative or qualitative outcome data were collected. No psychometric instruments assessing acute stress, peritraumatic dissociation, PTSD symptoms, heart rate variability, or cognitive performance were administered before, during, or after the intervention. The absence of such data means that the functional outcomes attributed to the protocol, including reduced helplessness, sustained cognitive engagement, and adaptive memory encoding, remain entirely inferential. The post-event observations reported are drawn from informal, unstructured conversations, lack any comparison condition, and are vulnerable to social desirability effects in a military context where acknowledgment of distress carries professional cost. The authors do not claim that the SIX Cs protocol caused the observed outcomes; the case demonstrates feasibility and theoretical coherence, not efficacy. Future work must move beyond the case report format toward prospective, controlled designs with standardized outcome measurement if the effectiveness of proactive SIX Cs implementation is to be established rather than inferred.

At the same time, it is worth acknowledging explicitly that what constitutes a methodological limitation in conventional research contexts may, in the setting of large-scale and unprecedented mass-casualty events, also reflect an unavoidable structural reality. Prospective data collection, whether quantitative or qualitative, is frequently not feasible during acute operational response to events of the scale and nature of the October 7 attack. In such contexts, retrospective, experience-based analyses conducted by those present during the event may represent among the only viable forms of scholarly contribution available. The present case report should therefore be understood not merely as a study constrained by the absence of systematic measurement, but as an illustration of the kind of theoretically grounded, practitioner-authored documentation that may be uniquely valuable precisely because it captures what controlled research designs cannot access: real-time, field-level implementation of a structured psychological intervention under conditions of maximum operational stress. Acknowledging this does not diminish the need for future prospective research; it contextualizes the present contribution within the broader landscape of knowledge generation in disaster mental health.

Beyond methodological constraints, the present case does not permit identification of the potential failure modes or contraindications of the proactive SIX Cs approach. The conditions that obtained in this case were unusually favorable: the team possessed years of shared operational experience, the commander had prior familiarity with the SIX Cs framework, and the mission, though psychologically extreme, proceeded without unexpected catastrophic deviation from the rehearsed protocol. Under different conditions, a newly formed unit, a commander without PFA training, or conditions that deviated sharply from those rehearsed, the same structured preparation could plausibly produce different outcomes, including false confidence, heightened anticipatory anxiety in trauma-vulnerable individuals, or the suppression of individual distress beneath the team's procedural scaffolding. A valid assessment of the protocol's generalizability requires not only replication under favorable conditions but deliberate investigation under conditions that may expose its boundary cases and failure modes.

The case is also situated within a highly specific operational, cultural, and historical context, an Israeli combat reserve unit responding to an unprecedented national mass-casualty event, and cannot be generalized to other organizational, cultural, or operational settings without independent verification. Additionally, it is not possible to isolate the contribution of the SIX Cs protocol from other protective factors present in this case, including pre-existing team cohesion, individual resilience, unit identity, and the meaning-making afforded by the national significance of the mission itself. These are not incidental confounds but substantive alternative explanations whose contribution to the observed outcomes cannot be dismissed on the basis of available evidence. Future research should employ prospective designs with standardized outcome measures, matched comparison conditions, and independent assessment to examine the efficacy of proactive SIX Cs implementation across diverse responder populations and contexts.

A productive direction for future work concerns the translation of the proactive SIX Cs framework from conceptual illustration into structured clinical and operational practice. The present case suggests that the model's three-phase structure, pre-exposure cognitive preparation, structured on-site execution, and post-mission cognitive closure, may be sufficiently specified to serve as the foundation for applied frameworks, commander-led training protocols, or implementation guidelines for first-responder organizations. Developing such resources would represent a meaningful next step in bridging the gap between theoretical formulation and field application, and would provide the structured basis upon which future empirical evaluation of the model's efficacy could be conducted. Collaborative development of these materials with operational commanders, mental health professionals, and first-responder training bodies would help ensure that the protocol is both psychologically grounded and operationally feasible across diverse deployment context.

## 5 Conclusion

This theory-driven case analysis illustrates the feasibility and theoretical coherence, of applying the SIX Cs Model as a proactive Psychological First Aid protocol in mass-casualty response operations. By systematically addressing possible cognitive and psychological precursors of ASR, anticipatory helplessness, cognitive disorganization, freezing, isolation, and memory fragmentation, before traumatic exposure, the structured pre-mission briefing extended the SIX Cs from a reactive to a prospective intervention modality. The subsequent evening AAR further extended the model into the post-exposure phase, potentially supporting adaptive cognitive integration and team cohesion. Together, these three phases constitute a comprehensive responder support structure grounded in cognitive PFA theory. Future work should examine the systematic implementation of proactive SIX Cs protocols in first-responder training curricula, military operational contexts, and mass-casualty preparedness programs, with attention to both performance outcomes and long-term psychological resilience.
